# Manipulation of Microbial Symbionts in *Bemisia tabaci* and *Trialeurodes vaporariorum* (Hemiptera: Aleyrodidae) Reveals Divergent Impacts on Insect Host Fitness and Plant Defense Modulation

**DOI:** 10.3390/insects17080775

**Published:** 2026-07-27

**Authors:** Marzieh Kashkouli, Jahangir Khajehali, Mohammad Mehrabadi

**Affiliations:** 1 Research Institute for Biotechnology and Bioengineering, Isfahan University of Technology, Isfahan 84156-83111, Iran; 2 Department of Plant Protection, College of Agriculture, Isfahan University of Technology, Isfahan 84156-83111, Iran; 3 Department of Entomology, Faculty of Agriculture, Tarbiat Modares University, Tehran 14115-336, Iran

**Keywords:** phylogenetic analysis, insect fitness trade-offs, jasmonic acid pathway, salicylic acid pathway, microbe-based pest control

## Abstract

Insect–microbe symbioses play pivotal roles in host ecology and plant–insect interactions, yet their species-specific functions in agricultural pests remain less understood. This study elucidates the functional role of symbiotic microbial communities in mediating insect host fitness and plant defense responses in two economically important whitefly species, *Bemisia tabaci* Gennadius and *Trialeurodes vaporariorum* Westwood (Hemiptera: Aleyrodidae). Using integrated molecular and physiological approaches, we characterized species-specific responses to antibiotic treatments (rifampicin and tetracycline) and their cascading effects on tripartite plant–insect–microbe interactions. In *B. tabaci*, antibiotic exposure induced significant depletion of the obligate symbiont *Portiera* and facultative *Rickettsia* (except for tetracycline-mediated *Portiera* proliferation), correlating with enhanced plant immune responses. In parallel, antibiotic treatments increased the titers of *Hamiltonella* and *Rickettsia* alongside constitutive plant defense suppression in *T. vaporariorum*, though tetracycline uniquely induced *AOS* expression elevation. Developmental assays revealed stage-specific vulnerabilities, with late nymphal and pupal stages showing high sensitivity to symbiont disruption, culminating in complete mortality within 40–50 d post-treatment. These findings show that microbial symbionts are essential to whitefly nutrition and evasion of plant anti-herbivore defenses. Our results provide a mechanistic basis for understanding symbiont-assisted invasion success in these whitefly species and underscore the potential of microbiome-targeted approaches for sustainable whitefly management.

## Simple Summary

Whiteflies rely on beneficial bacteria living inside their bodies to survive. Our research investigated the role of these bacterial partners in two major whitefly species. We used antibiotics to reduce or alter their bacterial communities and then studied the effects on the whiteflies and the plants they attacked. We found that disrupting these bacteria severely harmed the whiteflies’ development and survival, showing how essential the bacteria are. Furthermore, whiteflies with altered bacteria could no longer suppress the defense systems of the plants, making the plants more resistant. This discovery reveals that the bacteria are not just nutritional partners; they also help whiteflies evade plant defenses. Our work suggests that specifically targeting these beneficial bacteria could lead to new, environmentally friendly strategies to control these destructive agricultural pests.

## 1. Introduction

*Bemisia tabaci* Gennadius and *Trialeurodes vaporariorum* Westwood (Hemiptera: Aleyrodidae) rank among the most economically significant agricultural pests, inflicting damage through phloem feeding and vectoring plant viruses, ultimately reducing crop yields and compromising produce quality with cascading effects on market stability and food security [1,2]. While both are global pests, they often occupy distinct ecological niches; *B. tabaci* (sweet potato whitefly) is a highly invasive cryptic species complex dominant in open-field and greenhouse systems in warmer climates, whereas *T. vaporariorum* (greenhouse whitefly) is a well-defined species particularly adapted to protected cultivation in temperate regions [1,2]. Various managing methods have been explored, ranging from biological to physical control techniques, although their control is complicated due to their high reproductive rates, resistance to various control methods, and the challenges posed by their life cycles and behavior [3,4,5]. These factors contribute to the difficulty in managing these pests effectively and necessitate innovative integrated pest management strategies such as symbiont-based approaches.

Whiteflies maintain intricate symbiotic relationships with microorganisms that shape their ecological adaptability [6,7,8]. The obligate symbiont *Portiera* provides essential amino acids absent in phloem diets [9,10], while facultative symbionts like *Arsenophonus* supplement B vitamins (riboflavin, pyridoxal, folate) [11], acting as conditional mutualists that enhance host fitness under nutritional or environmental stress [12,13]. The other secondary symbiont, *Hamiltonella* contributes to vitamin and amino acid synthesis [14,15], whereas *Rickettsia* accelerates nymphal development, enhance overall reproductive success [16,17], confers resistance to insecticides and pathogenic bacteria [14,16].

Microbial symbionts of insects may actively modulate host plant physiology and suppress anti-herbivore defenses to enhance the fitness of their insect hosts [18]. These microbial partners employ multiple strategies to interfere with plant defense mechanisms, including the suppression of key defense pathways [19], manipulation of hormonal crosstalk between jasmonic acid (JA) and salicylic acid (SA) pathways [20], disrupting the plant’s ability to mount an effective defense against herbivores. These modifications not only weaken plant resistance but also enhance insect fitness by increasing reproductive output and survival rates [19]. Some insects further amplify this effect by introducing symbiotic bacteria or effector molecules directly into plant tissues via oral secretions, systemically impairing defense responses [20]. Symbionts may also contribute to detoxifying plant secondary metabolites or pesticides, providing an additional advantage to their hosts [19]. Collectively, these mechanisms underscore the sophisticated role of insect symbionts as covert mediators that reshape plant defense landscapes to favor herbivore success, highlighting their critical position within the broader framework of plant–insect–microbe interactions.

Recent advances in microbiome research have revealed that antibiotic treatments can serve as powerful experimental tools for probing symbiont function in insect pests [10,21]. Studies employing antibiotics such as tetracycline and rifampicin have demonstrated their efficacy in eliminating specific bacterial symbionts from whiteflies, thereby allowing researchers to examine the causal relationships between microbial communities and host phenotypic traits [22,23]. However, comparative studies examining differential symbiont dependence between *B. tabaci* and *T. vaporariorum* remain scarce, despite the potential for such investigations to reveal species-specific vulnerabilities. This study employs symbiont manipulations by antibiotics and fitness analysis to comparatively examine symbiont function in both whitefly species, with particular emphasis on how microbial associations influence host plant defense. By elucidating the mechanisms through which whitefly symbionts alter host plant physiology and insect fitness, this research contributes to the development of our growing understanding of tripartite plant–insect–microbe interactions.

## 2. Materials and Methods

### 2.1. Whitefly Collection and Rearing

Populations of *T. vaporariorum* and *B. tabaci* were collected in February 2023 from tomato greenhouses in Isfahan, Iran. After morphological confirmation of species identity [24,25,26,27], adult whiteflies were transferred to insect-proof cages (90 × 90 × 90 cm) containing tomato seedlings. The cages were housed in climate-controlled chambers under regulated environmental conditions: temperature (25 ± 2 °C), photoperiod (14:10 h light–dark), and relative humidity (60 ± 5%). The colonies were maintained for two generations under these standardized conditions prior to experimental use.

### 2.2. DNA Extraction, PCR Amplification, and Systematic Classification of Hosts and Symbionts

Genomic DNA was extracted from 20 adult whiteflies using the cetyltrimethylammonium bromide (CTAB) method [28], following surface sterilization (in 75% ethanol for 2 min and three sterile distilled water washes). The extracted DNA was evaluated for quality via agarose gel electrophoresis (1% gel, 90 V, 90 min), observed using Vilber gel documentation system (Vilber Lourmat, Collégien, France), and stored at −20 °C until further analysis.

For identification of whitefly species, *B. tabaci* biotypes, and the symbionts genus, PCR amplification was performed in 25 µL reactions containing approximately 50 ng DNA template, specific primers (Table 1), and PCR master mix (Ampliqon, Odense, Denmark). Whitefly species were identified using mitochondrial cytochrome c oxidase I (*COI*) gene markers (Table 1). To differentiate between *B. tabaci* biotypes B and Q, a microsatellite marker, Bem23-(GAA)31imp [29], was utilized in a standard PCR assay (Table 1). This method distinguishes the biotypes based on fragment length polymorphisms of the amplified PCR products. Gene markers for *16S rRNA* and *23S rRNA* were used to amplify endosymbionts such as *Portiera*, *Arsenophonus*, *Wolbachia*, *Hamiltonella*, *Rickettsia*, *Cardinium*, and *Fritschea* (Table 1). The thermal cycling protocol comprised an initial denaturation at 94 °C for 3 min; 30 cycles of denaturation (94 °C, 1 min), annealing (55 °C, 45 s), and extension (72 °C, 1 min); followed by a final extension at 72 °C for 10 min. Amplified products were checked by electrophoresis, purified using a Favorgen Gel Extraction Kit (Yekta Tajhiz Azma Co., Tehran, Iran), and sequenced via Sanger sequencing (Pishgam Biotechnology, Tehran, Iran).

The systematic positioning of host species and their associated symbionts was determined through molecular phylogenetic analysis. Initial sequence processing involved generating reverse complements of all reverse reads using BioEdit version 7.2 software [30]. Consensus sequences were created by aligning forward and reverse reads for each genetic marker using NCBI (National Center for Biotechnology Information) online platform. These consensus sequences were subsequently subjected to similarity searches against the GenBank database using the BLASTn algorithm (Basic Local Alignment Search Tool), with detailed evaluation of E-values, percentage identity, and query coverage for each target gene.

For comparative phylogenetic analysis, nucleotide sequences from taxonomically related organisms and appropriate outgroups were retrieved from the NCBI database in FASTA format. Multiple sequence alignment was performed using the ClustalW algorithm [31,32] implemented in MEGA11 software (version 11.0.13) [33], followed by manual curation of the alignments. Phylogenetic reconstruction was conducted using Maximum Likelihood (ML) method in MEGA11 (version 11.0.13). The optimal nucleotide substitution model for ML analysis was selected based on the lowest Bayesian Information Criterion (BIC) score. The phylogenetic trees were constructed with bootstrap replicates to assess the robustness of topological features.

### 2.3. Antibiotic Treatment Protocol

The antibiotic treatments were administered to synchronized adult whiteflies using an artificial diet-feeding system [34]. Whiteflies were confined in transparent plastic chambers (40 mm diameter × 85 mm height) equipped with a 4 mm ventilation hole. Each chamber was sealed with a double-layered Parafilm membrane containing a 200 µL aliquot of treatment solution. The artificial diet was composed of a 25% (*w*/*v*) sucrose solution supplemented with the respective antibiotics. A corresponding control group was maintained under identical environmental conditions, in which adult whiteflies were provided with the same artificial diet (25% sucrose solution) without antibiotic supplementation.

For the antibiotic treatments, either rifampicin or tetracycline hydrochloride (Hakim Pharmaceutical Co., Tehran, Iran) was administered at a concentration of 50 μg/mL. These treatments lasted for 48 h, with the diet solution being replaced after 24 h. The choice of antibiotics, their concentrations, treatment durations, and specific applications was based on empirical evidence from previous studies [10,21,22,23,34,35], which confirmed their efficacy while ensuring no antifeedant effects. Furthermore, antibiotic selection adhered to the criterion that assay mortality did not exceed 15–25%, as established through pretreatment assessments. These evaluations revealed that higher concentrations and prolonged treatment durations led to mortality rates beyond acceptable thresholds. All experimental procedures were conducted under strictly controlled environmental conditions maintained at 25 ± 2 °C, 60 ± 5% relative humidity, with a 14:10 h light: dark photoperiod. Following the treatment period, surviving adult whiteflies were collected for subsequent studies.

### 2.4. Quantification of Symbiont Abundance

The relative abundance of bacterial symbionts in different treatment groups was assessed through quantitative PCR (qPCR) analysis. All reactions were performed on a StepOne Real-Time PCR system (Applied Biosystems, San Francisco, CA, USA) using 20 µL reaction volumes containing SYBR Green High Rox master mix (Ampliqon, Odense, Denmark), nuclease-free water, specific primer pairs (Table 2), and template DNA extracted from pools of 20 adult females per treatment. Sterile water served as the negative control in all assays. We targeted four key symbionts: the obligate symbiont *Portiera* (*16S rRNA*), and three facultative symbionts-*Arsenophonus* (*23S rRNA*), *Hamiltonella* (*16S rRNA*), and *Rickettsia* (*gltA*). The host *β-actin* of *B. tabaci* and *NADH* of *T. vaporariorum* were amplified as an endogenous reference for normalization (Table 2). The standardized qPCR protocol consisted of an initial enzyme activation at 95 °C for 10 min, followed by 40 cycles of denaturation (95 °C, 15 s), annealing (60 °C, 30 s), and extension (72 °C, 30 s). Each run concluded with a melt curve analysis (60–95 °C) to confirm amplification specificity. All samples were run in five (*B. tabaci* symbionts) and four (*T. vaporariorum* symbionts) replications, and cycle threshold (Ct) values were recorded for subsequent analysis.

Quantification of relative symbiont abundance was performed using the ΔΔCt method [36], with normalization to the reference gene and comparison to control samples. Statistical analysis involved one-way ANOVA followed by Tukey’s post hoc test (α = 0.05) to identify significant differences in symbiont abundance across treatment groups. All statistical computations were performed using SPSS software (version 25, IBM Corp., Armonk, NY, USA), while graphical representations were generated using GraphPad Prism (version 9.0). This comprehensive approach allowed for precise quantification of treatment-induced changes in symbiont populations while controlling for potential technical variability.

### 2.5. Fitness and Biological Trait Analyses

Individuals from control and antibiotic treatments for each whitefly species were subjected to fitness evaluations. The adults were allowed to lay eggs and the newly emerged hatchlings were monitored daily until either mortality or adulthood. Key biological parameters, including developmental duration and survival rates, were recorded at 24 h intervals until the death of insects. Stage-specific survival rates (defined as the probability that a neonate will survive to stage j, but it may die in stage j) and stage composition (assessed at 10 d intervals) were quantified. Statistical comparisons were conducted using the Fisher-Freeman-Halton exact test in SPSS. All data visualizations were generated using GraphPad Prism.

### 2.6. Evaluation of Plant Defense Responses to Insect Feeding

To assess the potential role of whitefly endosymbionts in modulating plant defense responses, we conducted feeding experiments comparing symbiont-manipulated and control whitefly populations. Two hundred adult insects from each treatment group (symbiont-manipulated and untreated controls) were subjected to a 2 h starvation period prior to being released onto caged tomato plants. All infested plants were maintained for one complete whitefly generation under standardized environmental conditions in controlled-climate growth chambers to allow for potential defense induction.

For analysis of defense-related gene expression profiles, total RNA was isolated from plant tissues using an optimized lithium chloride (LiCl) precipitation protocol [37]. The extraction buffer consisted of 2% CTAB in 100 mM Tris-HCl (pH 8.0), 1.7 M NaCl, and 20 mM EDTA, prepared under RNase-free conditions. Approximately 50–100 mg of tomato leaf tissue was flash-frozen in liquid nitrogen and mechanically homogenized using a pre-chilled mortar and pestle. Tissue lysates were incubated in CTAB buffer supplemented with 2-mercaptoethanol at 60 °C for 20 min, followed by chloroform: isoamyl alcohol (24:1) extraction. RNA was precipitated overnight at −20 °C using 10 M LiCl, with subsequent washing in 70% ethanol. RNA integrity was verified by electrophoretic separation on 1% agarose gels.

For transcriptional profiling, first-strand cDNA was synthesized from total RNA using reverse transcriptase. Quantitative real-time PCR analyses were performed in triplicate as previously described. We targeted key jasmonic acid (JA) pathway marker *AOS* (allene oxide synthase), and salicylic acid (SA)-responsive *PR-1* (pathogenesis-related protein 1), with *ubiquitin* serving as the endogenous control [38]. Amplification conditions consisted of initial denaturation (95 °C, 15 min) followed by 40 cycles of 95 °C for 15 s, 60 °C for 20 s, and 72 °C for 32 s. Post-amplification melt curve analysis confirmed reaction specificity. Relative gene expression was calculated using the 2^−ΔΔCt^ method [36], with statistical analysis performed using SPSS software and visualization performed in GraphPad Prism.

## 3. Results

### 3.1. Molecular Characterization of the Whitefly and Their Associated Bacterial Symbionts

The mitochondrial *COI* gene fragments were amplified (Supplementary Figure S1A) and their sequences confirmed the identity of both whitefly species, with *T. vaporariorum* and *B. tabaci* sequences deposited in GenBank under accession numbers PP577665 and PP577664, respectively. Additionally, biotype identification of *B. tabaci* was performed using microsatellite markers, revealing that the specimens belonged to biotype B, as confirmed by the PCR fragment size of 200 bp (Supplementary Figure S1B). Our analysis revealed a consistent symbiotic profile across both species, consisting of the obligate primary symbiont *Portiera* along with three facultative secondary symbionts: *Hamiltonella, Arsenophonus*, and *Rickettsia*. All obtained nucleotide sequences have been archived in NCBI GenBank to facilitate future research, with *T. vaporariorum* symbionts deposited as PP537908 *(Portiera 16S rRNA*), PP537907 (*Hamiltonella 16S rRNA*), PP536068 (*Arsenophonus 23S rRNA*), and PP537909 (*Rickettsia 16S rRNA*), while *B. tabaci* symbionts were assigned accession numbers PP537904 (*Portiera*), PP537905 (*Hamiltonella*), PP536067 (*Arsenophonus*), and PP537906 (*Rickettsia*).

The phylogenetic relationships of insect *COI, Portiera, Hamiltonella, Rickettsia 16S rRNA,* and *Arsenophonus 23S rRNA* sequences were reconstructed using Maximum Likelihood (ML) method. Phylogenetic analysis of whitefly *COI* sequences consistently resolved *T. vaporariorum* and *B. tabaci* as distinct monophyletic groups with strong nodal support and *Trialeurodes ricini Misra (Hemiptera: Aleyrodidae)* serving as an effective outgroup (Supplementary Figure S1C).

The *Portiera* phylogenies revealed well-supported clades across all reconstruction methods (Supplementary Figure S2A). Similarly, *Hamiltonella* strains formed distinct, well-supported clades specific to each host species (Supplementary Figure S2B). The *Arsenophonus* phylogeny also demonstrated strong host-associated grouping, with three well-supported clusters corresponding to their hosts (Supplementary Figure S2C). The *Rickettsia* phylogeny exhibited characteristically long branches and strong host-associated clustering, consistent with the known accelerated evolutionary rate of this genus (Supplementary Figure S2D).

### 3.2. Quantification of Symbiont Abundance

**Figure 1:**
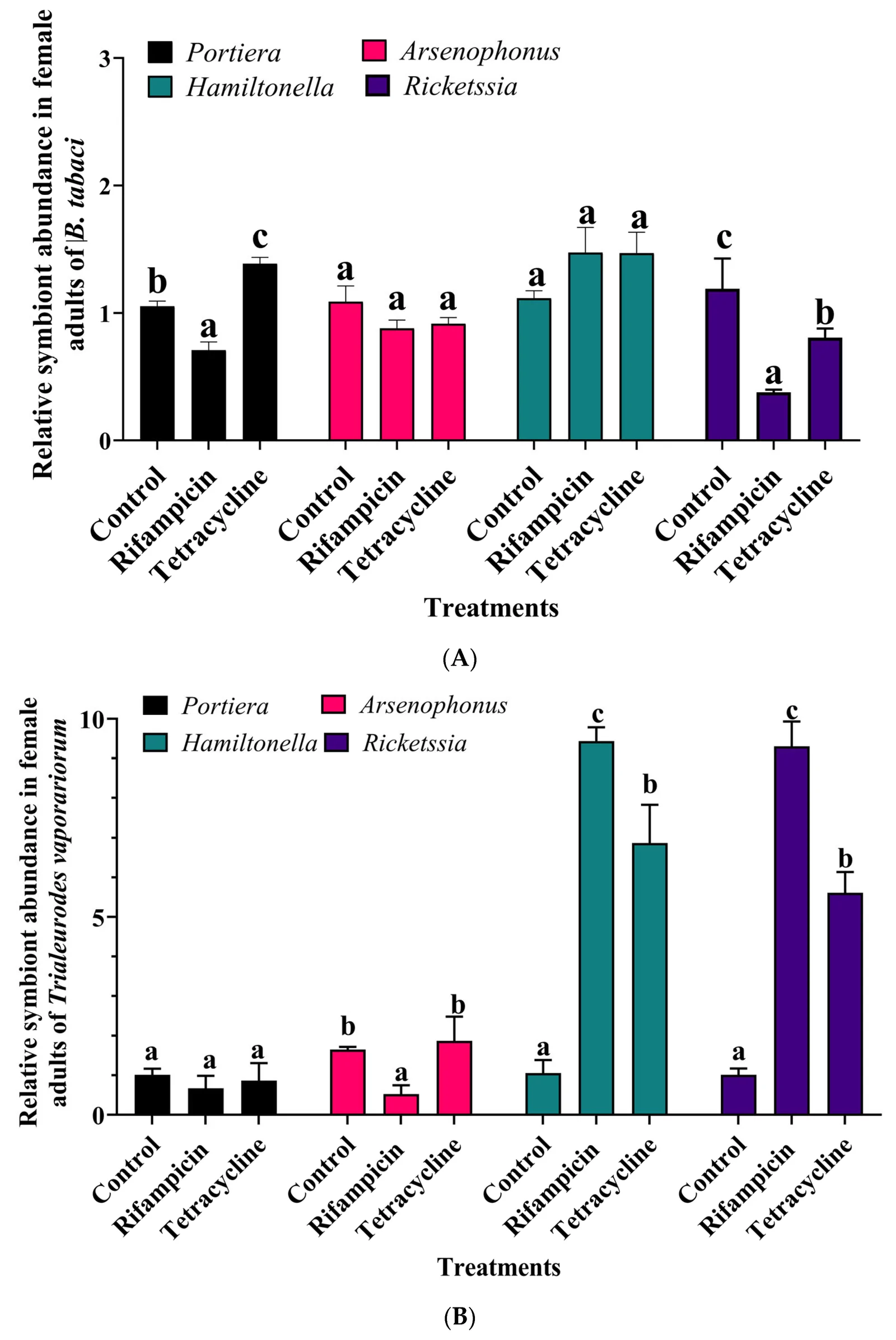
Differential effects of antibiotic treatments (tetracycline and rifampicin) on symbiont abundance in whitefly species. (**A**) *Bemisia tabaci* and (**B**) *Trialeurodes vaporariorum* showing relative quantification (qPCR) of primary (*Portiera*) and secondary (*Arsenophonus*, *Hamiltonella*, *Rickettsia*) symbionts following exposure to rifampicin, tetracycline, or control treatments. Data represent mean ± SEM (*n* = 3 biological replicates). Lowercase letters indicate statistically significant differences among treatments (*p* < 0.05, Tukey’s post hoc test).

Quantitative PCR analysis revealed distinct patterns of symbiont modulation in *B. tabaci* following antibiotic treatments (Figure 1A). *Portiera* abundances showed significant differences between treatments (F (2,12) = 40.32; *p* ≤ 0.001). Rifampicin treatment caused significant reduction in this obligate symbiont. Intriguingly, tetracycline treatment induced paradoxical increases in *Portiera* abundance suggesting complex inter-symbiont dynamics under antimicrobial pressure. In contrast, *Arsenophonus* (F (2,12) = 0.74; *p* = 0.498) and *Hamiltonella* (F (2,12) = 1.82; *p* = 0.203) titers remained stable across all treatment regimens, demonstrating exceptional resistance to antibiotic perturbation varied significantly among different antibiotic treatments. *Rickettsia* showed vulnerability to rifampicin and tetracycline significantly (F (2,12) = 40.05; *p* ≤ 0.001), further underscoring differential symbiont-specific responses to antimicrobial agents (Figure 1A).

*T. vaporariorum* displayed fundamentally distinct symbiont population dynamics under antibiotic treatment compared to *B. tabaci* (Figure 1B). Quantitative analysis revealed no significant perturbation in *Portiera* titers across all treatment groups (F (2,9) = 0.82; *p* = 0.472), indicating remarkable stability of this obligate symbiont. In contrast, *Arsenophonus* populations exhibited significant depletion following rifampicin exposure (F (2,9) = 10.84; *p* = 0.004). Most strikingly, we documented significant increases in *Hamiltonella* (F (2,9) = 143.47; *p* ≤ 0.001) and *Rickettsia* (F (2,9) = 221.89; *p* ≤ 0.001) densities under antimicrobial pressure, with effect sizes ranging from 1.5- to 2.3-fold relative to controls (Figure 1B).

### 3.3. Fitness and Biological Analyses of Symbiont-Manipulated Whiteflies

**Figure 2:**
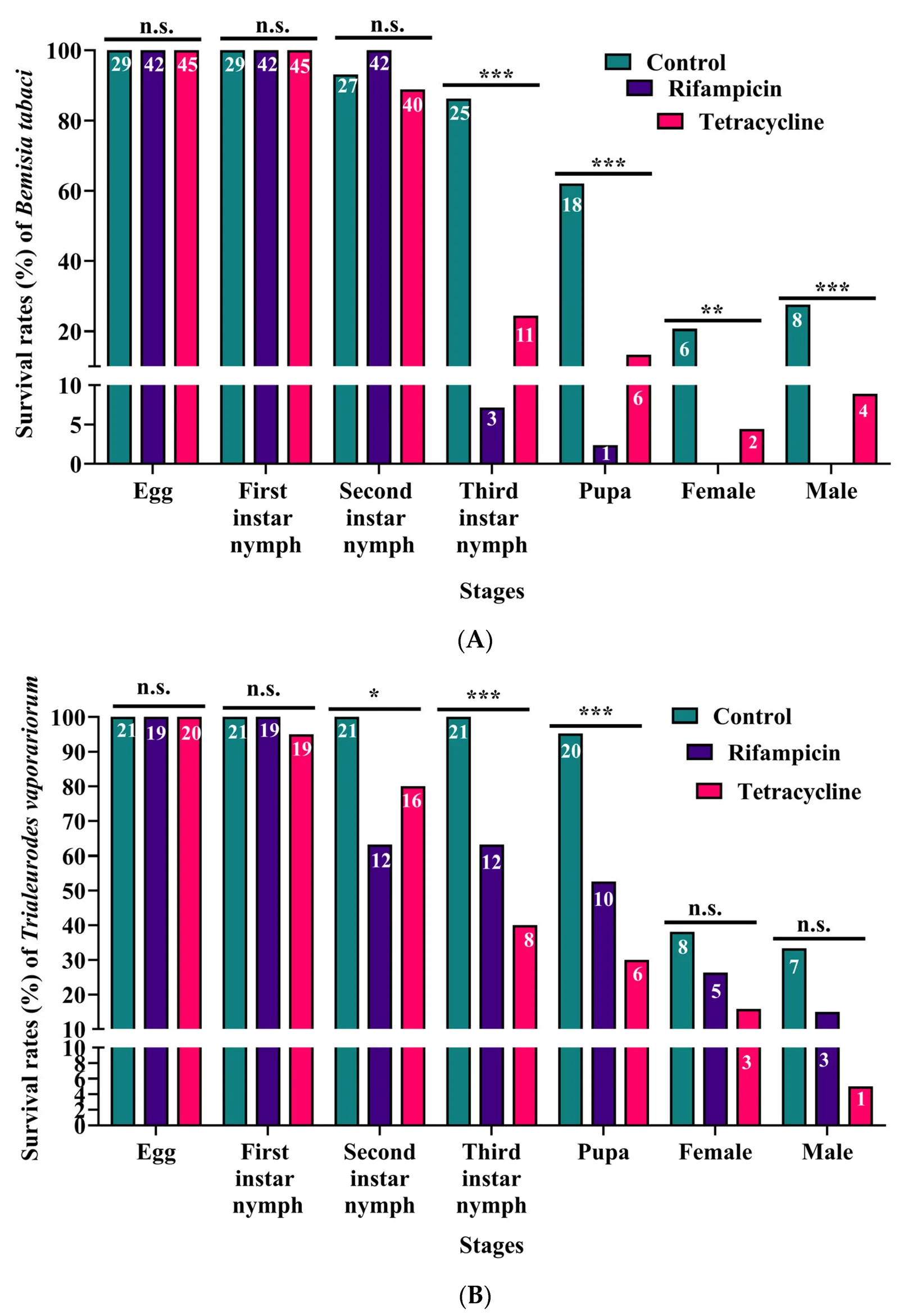
Impact of antibiotic treatments (tetracycline and rifampicin) on the survival rates of whiteflies, *Bemisia tabaci* (**A**) and *Trialeurodes vaporariorum* (**B**). The number of individuals entered each stage is presented on each column. Survival rates were compared for each stage using Fisher-Freeman-Halton exact probability test: n.s., not significant, *: *p* < 0.05, **: *p* < 0.01, and ***: *p* ≤ 0.001.

Antibiotic treatments altered the survival rates of both *B. tabaci* and *T. vaporariorum*, compared to the untreated control group. In *B. tabaci*, survival did not differ significantly among treatments during the egg, first-instar nymph (N1), or second-instar nymph (N2) stages (*p* > 0.05) (Figure 2A). However, pronounced differences emerged in later developmental stages, including third-instar nymphs (N3) (*p* ≤ 0.001), pupae (*p* ≤ 0.001), females (*p* = 0.002), and males (*p* ≤ 0.001) (Figure 2A). Rifampicin exposure caused the most severe survival reduction in these stages, whereas tetracycline had a comparatively moderate but still significant effect.

In *T. vaporariorum,* no significant survival differences were detected in egg, N1, or adult stages (*p* > 0.05) (Figure 2B). However, antibiotic treatments significantly impacted N2 (*p* = 0.032), N3 (*p* ≤ 0.001), and pupal (*p* ≤ 0.001) stages (Figure 2B). Unlike *B. tabaci*, tetracycline exerted a stronger negative effect on survival in *T. vaporariorum* than rifampicin, except in the N2 stage, where rifampicin showed greater lethality.

**Figure 3:**
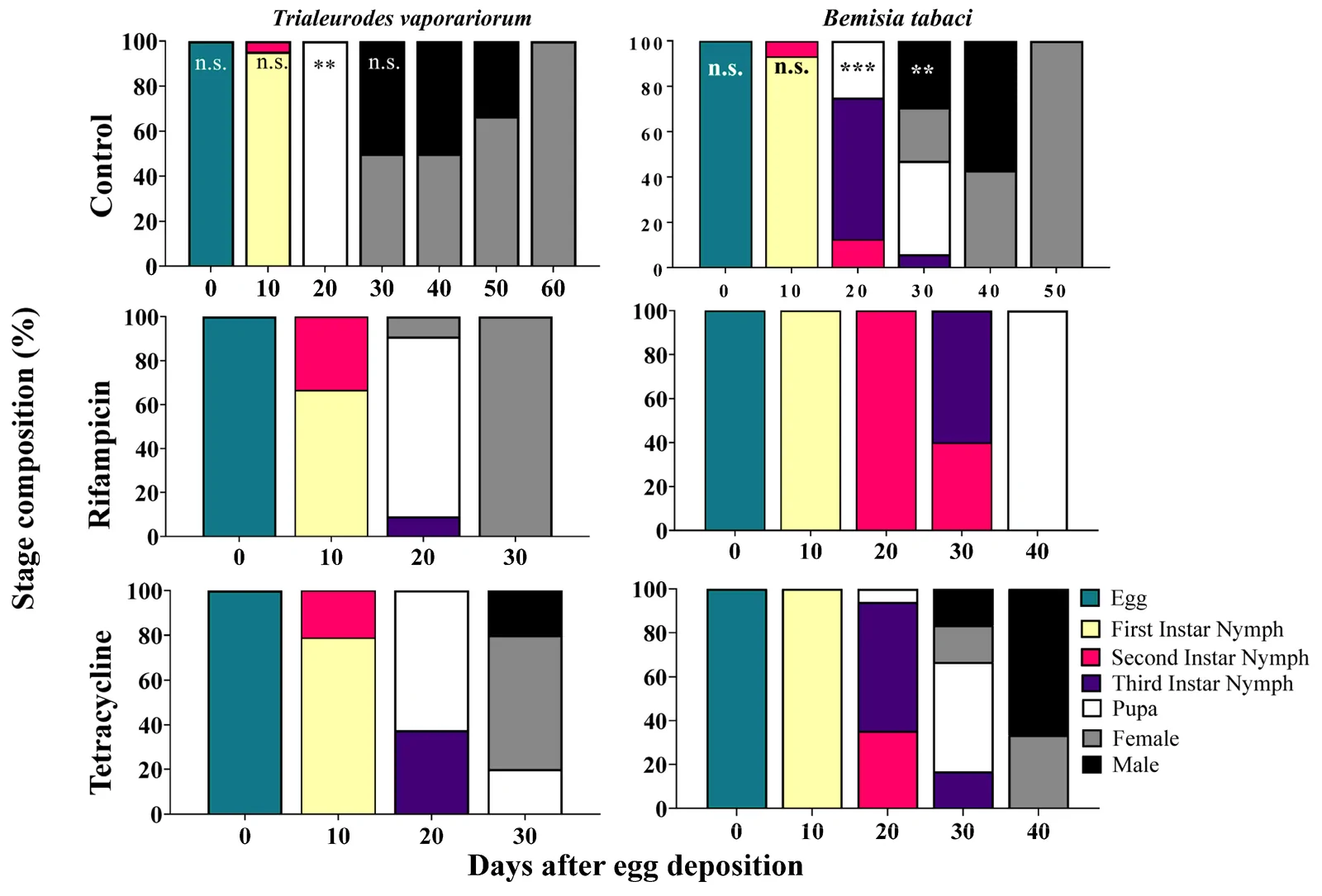
Growth profiles of whiteflies, *Bemisia tabaci* and *Trialeurodes vaporariorum* treated with different antibiotic treatments (tetracycline and rifampicin). Stage compositions were compared for each whitefly species and time interval using Fisher-Freeman-Halton exact probability test: n.s., not significant, **: *p* < 0.01, and ***: *p* ≤ 0.001.

Antibiotic exposure disrupted and shortened the developmental progression of both whitefly species. In *B. tabaci*, antibiotic treatments induced pronounced developmental delays, with a significantly higher proportion of individuals remaining in early nymphal stages at both 20 d (*p* ≤ 0.001) and 30 d (*p* = 0.008) post-egg deposition compared to controls (Figure 3). Notably, no surviving individuals were observed in either tetracycline- or rifampicin-treated groups by the 40 d and further timepoints. Similarly, in *T. vaporariorum*, both antibiotics caused significant prolongation of nymphal durations at the 20 d interval (*p* = 0.009). Mirroring observations in *B. tabaci*, complete mortality was recorded in antibiotic-treated groups before day 50 (Figure 3).

### 3.4. Differential Induction of Plant Defense Pathways by Whiteflies with Modified Symbiotic Communities

**Figure 4:**
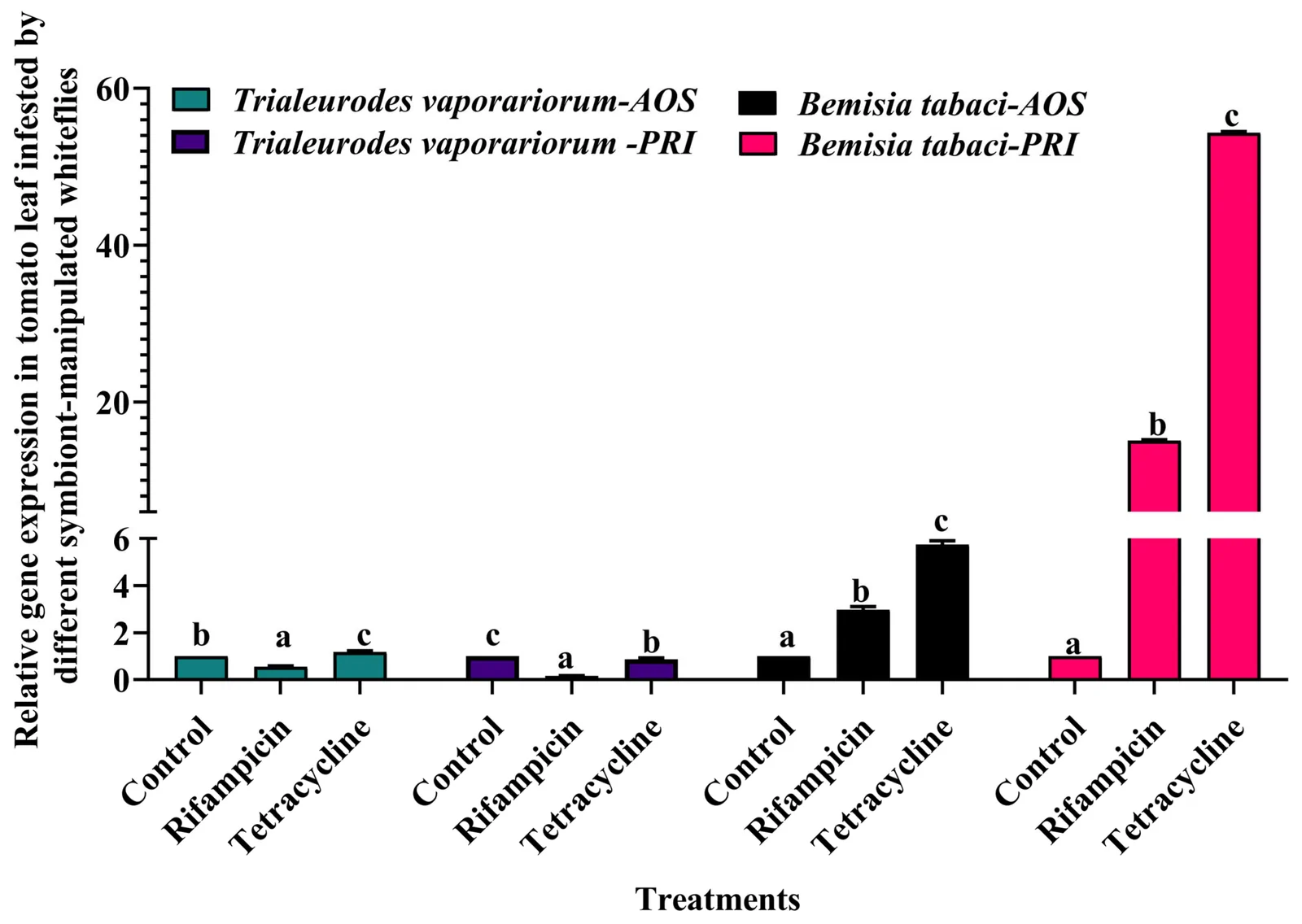
Species-specific modulation of tomato defense responses by symbiont-manipulated whiteflies, *Bemisia tabaci* and *Trialeurodes vaporariorum*. Jasmonic acid (*AOS*) and salicylic acid (*PR-1*) pathway genes induce differentially following antibiotic treatments. Data represent mean ± SEM (*n* = 3 biological replicates). Lowercase letters indicate statistically significant differences among treatments (*p* < 0.05, Tukey’s post hoc test).

Our investigation into how symbiotic community manipulation affects plant defense responses revealed significant alterations (*p* ≤ 0.001) in jasmonic acid (JA) and salicylic acid (SA) pathway activation following of both whiteflies’ feeding (Figure 4). Notably, *T. vaporariorum* carrying rifampicin-altered symbionts induced significant suppression of the JA pathway marker *AOS*, whereas tetracycline-treated counterparts elicited mild upregulation. Both antibiotic treatments consistently downregulated expression of the SA marker *PR-1* in *T. vaporariorum*-infested plants, with rifampicin causing particularly strong suppression compared to tetracycline treatment (Figure 4).

In contrast, *B. tabaci* with modified symbiotic communities triggered robust activation of both defense pathways. Plants infested with rifampicin-treated *B. tabaci* showed 2.97-fold induction of *AOS* and 14.94-fold upregulation of *PR-1*, while tetracycline-altered symbionts resulted in even more pronounced effects, 5.51-fold and 54.32-fold increases for *AOS* and *PR-1*, respectively (Figure 4). These dramatic differences highlight the critical role of intact symbiotic communities in modulating plant defense responses to whitefly infestation.

## 4. Discussion

Our study demonstrates that symbiotic microbial communities play a fundamental role in mediating whitefly biology and plant defense responses, revealing distinct evolutionary trajectories between *B. tabaci* and *T. vaporariorum*. The consistent genetic differentiation between *T. vaporariorum* and *B. tabaci* validates *COI* as a reliable molecular marker for species delineation in whiteflies. Phylogenetic analyses confirm strong evolutionary conservation of primary symbionts, while secondary symbionts exhibit host-specific association patterns. Notably, host-specific clustering of *Hamiltonella* and *Arsenophonus* strains supports coadaptation dynamics, aligning with established models of vertical transmission fidelity [39,40,41]. The accelerated molecular evolution observed in *Rickettsia*, evidenced by elongated branch lengths, reflects genomic reduction and intracellular lifestyle constraints [42,43]. Together, these findings underscore how host ecology drives symbiont evolutionary trajectories, with implications for understanding insect–microbe coevolution.

Species-specific responses to antibiotic treatments reveal critical windows of symbiont dependency during whitefly development [44]. Notably, *Portiera* in *T. vaporariorum* remains stable under antibiotic treatment consistent with its obligate, gnomically integrated role [9], contrasting sharply with its sensitivity in *B. tabaci*, suggesting fundamental differences in host-symbiont coevolution. The unexpected increase in *Portiera* abundance observed in tetracycline-treated *B. tabaci* may reflect either compensatory host mechanisms or competitive release dynamics. This phenomenon can be attributed to the ecological competition between primary (*Portiera*) and secondary symbionts, which co-localize in bacteriocytes and compete for limited cellular space and host-derived nutritional resources [10]. Tetracycline-induced suppression of secondary symbionts likely alleviated this competition, thereby permitting *Portiera*—as an obligate nutritional mutualist—to expand its population through enhanced resource acquisition.

Notably, rifampicin treatment significantly declined *Portiera* abundance in *B. tabaci*, and *Arsenophonus* abundance in *T. vaporariorum*, aligning with previous reports of partial inactivation under rifampicin exposure [23,45]. Detailed studies specifically focusing on the mechanisms by which rifampicin affects the bacteria are limited; however, some studies indicated that rifampicin inhibits bacterial RNA polymerase (RNAP), which is essential for DNA transcription [46]. In addition, *B. tabaci* populations showed significant *Rickettsia* depletion following antibiotic treatment. The effects of antibiotics on the *Rickettsia* symbiont in whiteflies, particularly through rifampicin treatment, demonstrate significant impacts on symbiont abundance. Specifically, rifampicin treatment leads to a marked reduction in *Rickettsia* density, although it does not completely eliminate it, especially in the offspring of treated adults, with significant reductions observed in the F1 generation [46,47]. In *T. vaporariorum*, *Rickettsia* and *Hamiltonella* exhibited significant population increases following rifampicin and tetracycline administration. Rifampicin-induced microbial community disruption appears to facilitate proliferation of these symbionts through competitive release, as sensitive bacterial populations are suppressed. Similarly, tetracycline (a broad-spectrum protein synthesis inhibitor [48]) enhanced *Hamiltonella* and *Rickettsia* abundance, likely through analogous competitive mechanisms.

The observed differential effects of antibiotic treatments on *B. tabaci* and *T. vaporariorum* provide compelling evidence for the critical role of microbial symbionts in whitefly development and survival. The species-specific responses to antibiotic treatments are particularly noteworthy. While rifampicin showed greater lethality in *B. tabaci*, tetracycline had more pronounced effects in *T. vaporariorum*. The developmental reduction and mortality observed in rifampicin-treated *B. tabaci*, correlate with the concomitant reduction in *Portiera* endosymbiont titers, suggesting a causal relationship between symbiont depletion and host fitness decline. In addition, our results demonstrate stage-specific vulnerabilities to symbiont disruption, with late nymphal and pupal stages showing particular sensitivity across both species. The complete mortality in treated groups before reaching 40–50 d suggests that microbial contributions become increasingly vital as whiteflies progress through their developmental cycle, likely due to symbiont-mediated nutritional provisioning [42,49] or involvement in hormone biosynthesis [17].

Our investigation of plant defense pathways reveals that symbiotic microbial communities serve as critical biological interfaces mediating plant–insect interactions. Distinct JA/SA modulation patterns between whitefly species reflect evolutionary adaptations for host defense evasion. In *B. tabaci*, antibiotic-induced symbiont depletion (notably of *Portiera* and *Rickettsia*, except for increased *Portiera* levels under tetracycline treatment) correlates with enhanced plant immune responses, suggesting these symbionts naturally suppress host defenses to benefit their insect hosts. On the other hand, *T. vaporariorum* demonstrates constitutive suppression of plant defenses following antibiotic treatments (with the exception of elevated *AOS* expression in tetracycline-treated specimens). The observed increases in *Hamiltonella* and *Rickettsia* titers following antibiotic treatments further support their role in defense modulation of *T. vaporariorum* host plant.

These findings align with established mechanisms of symbiont-mediated defense manipulation. For instance, *Rickettsia* symbionts in *B. tabaci* whiteflies enhance SA-mediated defenses while concurrently suppressing JA-dependent resistance pathways, a key component of plant anti-herbivore defense, thereby increasing whitefly fecundity and survival [50]. This JA pathway repression, mediated by symbiont-derived salivary effectors or hormonal crosstalk, has been similarly documented in plants infested with *B. tabaci* [51,52]. Furthermore, the whitefly-associated symbiont *H. defensa* suppresses induced defenses in tomato plants, which was mediated by a <3 kDa non-proteinaceous salivary effector molecule impairing herbivory recognition and facilitating infestation [38]. Future work should focus on identifying the specific effector molecules responsible for this phenomenon and functionally validating their role in manipulating plant JA/SA pathways. A key testable hypothesis is that the elevated titers of *Hamiltonella* and *Rickettsia* in *T. vaporariorum* result in a greater abundance of such effectors in whitefly saliva, leading to more potent suppression of plant defenses.

The nymphal and adult *B. tabaci* actively alter SA and JA levels in host plants, with symbionts potentially exacerbating this effect to further weaken anti-herbivore responses [51,52]. Notably, *Rickettsia*-infected plants exhibit a trade-off, displaying enhanced resistance to fungal (*Verticillium* spp.) and viral pathogens (e.g., Tomato yellow leaf curl virus, TYLCV) due to SA pathway activation, yet simultaneously increasing susceptibility to insect herbivores [50]. Additionally, *Rickettsia* can persist in tomato phloem for extended periods (>30 d), continuously modifying defense-related gene expression (e.g., upregulating *TGA 2.1* and *VRP* genes) to promote whitefly performance [50]. Beyond direct defense manipulation, *Hamiltonella* infection in *B. tabaci* contributes to maintaining a balanced host sex ratio, ensuring the stability of symbiont-mediated defense suppression across generations [14]. Collectively, these interactions underscore the dual role of insect symbionts in simultaneously undermining plant resistance to herbivores while enhancing pathogen defenses, revealing intricate ecological and evolutionary dynamics that could inform novel microbiome-based pest management strategies.

Future research should prioritize mechanistic studies of symbiont effectors (using multi-omics tools), field validation of microbial disruption strategies, and ecological risk assessments of symbiont transfer and resistance evolution. By bridging fundamental knowledge of tripartite plant–insect–microbe interactions with applied pest control solutions, this work lays the foundation for developing ecologically balanced, microbiome-based agricultural interventions while emphasizing the need for long-term studies to evaluate unintended ecological consequences.

## Figures and Tables

**Table 1:** Oligonucleotide primers used for PCR-based identification of whitefly and symbiont species.

Targeted Genes	Primer Names	Expected Amplicon Sizes (bp)	Primer Sequences (5′–3′)
*Trialeurodes vaporariorum* and *Bemisia tabaci mtCO1*	C1-J-2195	850	TTGATTTTTTGGTCATCCAGAAGT
	L2-N-3014		TCCAATGCACTAATCTGCCATATTA
*B. tabaci* B/Q biotypes micro-sattelite	Bem23-F	B biotype: 200 bp Q biotype: 400 bp	CGGAGCTTGCGCCTTAGTC
	Bem23-R		CGGCTTTATCATAGCTCTCGT
*Portiera 16S rRNA*	28F	1000–1100	TGCAAGTCGAGCGGCATCAT
	1098R		AAAGTTCCCGCCTTATGCGT
*Arsenophonus 23S rRNA*	Ars23S.1	600	CGTTTGATGAATTCATAGTCAAA
	Ars23S.2		GGTCCTCCAGTTAGTGTTACCCAAC
*Wolbachia 16S rRNA*	Wol16S-f	600	CGGGGGAAAAATTTATTGCT
	Wol16S-r		AGCTGTAATACAGAAAGTAAA
*Hamiltonella 16S rRNA*	Ham-F	1000	TGAGTAAAGTCTGGGAATCTGG
	Ham-R		CCCGGGAACGTATTCACCGTAG
*Rickettsia 16S rRNA*	RB_F	900	GCTCAGAACGAACGCTATC
	RB_R		GAAGGAAAGCATCTCTGC
*Cardinium 16S rRNA*	CFB_F	400	GCGGTGTAAAATGAGCGTG
	CFB_R		ACCTMTTCTTAACTCAAGCCT
*Fritschea 23S rRNA*	U23F	600	GATGCCTTGGCATTGATAGGCGATGAAGGA
	23SIGR		TGGCTCATCATGCAAAAGGCA

**Table 2:** Oligonucleotide primers used for quantification of symbiont species by quantitative PCR (qPCR).

Targeted Genes	Primer Names	Expected Amplicon Sizes (bp)	Primer Sequences (5′–3′)
*Bemisia tabaci Actin*	Bactin-F	130	TCTTCCAGCCATCCTTCTTG
	Bactin-R		CGGTGATTTCCTTCTGCATT
*Portiera* symbiont of *Bemisia tabaci 16S rRNA*	Bport-F	200	GTGGGGAATAACGTACGG
	Bport-R		CTCAGTCCCAGTGTGGCTG
*Portiera* symbiont of *Trialeurodes vaporariorum 16S rRNA*	Tport-F	168	GTAATACGGAGGGTGCAAGC
	Tport-R		ATTTCACCGCTACACCTGGA
*Trialeurodes vaporariorum NADH*	Tnadh-F	99	GCGCTTTGCTTTACCTTTGT
	Tnadh-R		GGTGGTCGGCAAGATTTGTT
*Arsenophonus* *23S rRNA*	qArs-F	174	ATGGTGCCGTAACTTCAGGA
	qArs-R		TAACCTTACAGCACCTGGCA
*Rickettsia* *gltA*	qRick-F	200	TGGTATTGCATCGCTTTGGG
	qRick-R		TTTCTTTAAGCACTGCAGCACG
*Hamiltonella* *16S rRNA*	qHam-F	243	GCATCGAGTGAGCACAGTTT
	qHam-R		TATCCTCTCAGACCCGCTAGA

## Data Availability

The data presented in this study are available on request from the corresponding author. The data are not deposited in a public repository due to their integral role in the authors’ active and forthcoming research projects.

## Supplementary Materials

The following supporting information can be downloaded at: https://www.mdpi.com/article/10.3390/insects17080775/s1, Figure S1: Detection of whiteflies, (A) Amplification of the whitefly *COI* gene (B) Detection of *B. tabaci* biotype, the electrophoresis gel contains positive control (C+) and four replications (R1–R4), (C)Phylogenetic reconstruction of whitefly *COI* gene sequences using Maximum Likelihood analysis employing the Hasegawa-Kishino-Yano (HKY) model with invariant sites. Numbers at nodes represent bootstrap support values. *T. ricini* was included as an outgroup; Figure S2: Phylogenetic reconstruction of *Portiera* (A)*, Hamiltonella, Arsenophonus* (B), and *Rickettsia* endosymbionts of *Bemisia tabaci* (C) and *Trialeurodes vaporariorum* (D) using Maximum Likelihood (ML) analysis. Numbers at nodes represent bootstrap support values.
