# Supplementary material for: Manipulation of Microbial Symbionts in Bemisia tabaci and Trialeurodes vaporariorum (Hemiptera: Aleyrodidae) Reveals Divergent Impacts on Insect Host Fitness and Plant Defense Modulation
Source: Insects. 2026 Jul 27;17(8):775. doi: 10.3390/insects17080775 (PMC13513110; doi:10.3390/insects17080775)
Supplement: Supplementary file 1 [file insects-17-00775-s001.zip › insects-3863625-supplementary.pdf]

Manipulation of microbial symbionts in *Bemisia tabaci* and *Trialeurodes vaporariorum* (Hemiptera: Aleyrodidae) reveals divergent impacts on insect host fitness and plant defense modulation

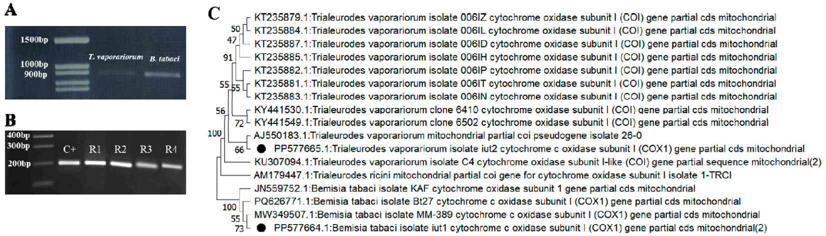

Supplementary Figure S1

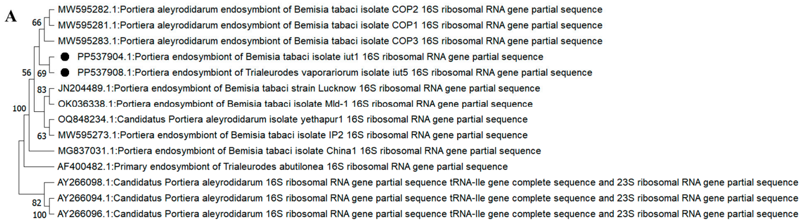

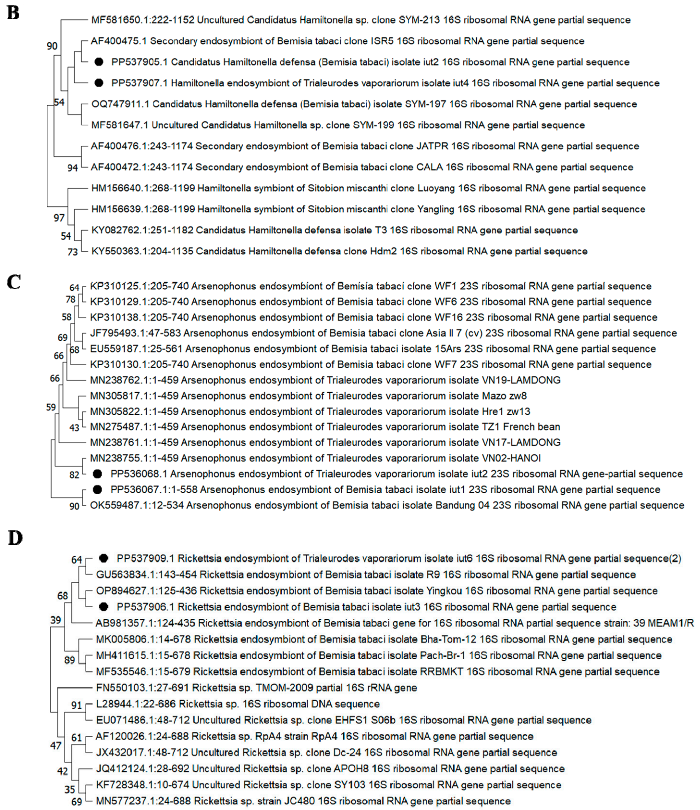

Supplementary Figure S2
